# Plasma C4 level was associated with mortality, cardiovascular and cerebrovascular complications in hemodialysis patients

**DOI:** 10.1186/s12882-022-02829-0

**Published:** 2022-06-29

**Authors:** Zheyu Xing, Yaqin Wang, Kunjing Gong, Yuqing Chen

**Affiliations:** 1 Renal Division, Peking University First Hospital, Beijing, China; 2 Institute of Nephrology, Peking University, Beijing, China; 3 Key Laboratory of Renal Disease, Ministry of Health of China, Beijing, China; 4 Key Laboratory of CKD Prevention and Treatment, Ministry of Education of China, Beijing, China

**Keywords:** Hemodialysis, Complement factor 4, Complement activation, Cardiovascular and cerebrovascular diseases, Prognosis, All-cause mortality

## Abstract

**Background:**

Patients on maintenance hemodialysis (HD) exhibit a high risk of death, cardiovascular and cerebrovascular diseases (CCDs). Previous studies indicated complement activation associated with the increased risk of cardiovascular diseases in HD patients. This study aimed to explore whether the critical complement factors were associated with the adverse outcomes in HD patients.

**Methods:**

A total of 108 HD patients were included and followed up for 52 months. The baseline clinical characteristics and plasma C3c, C1q, CFH, CFB, C4, MAC, C5a, C3a and MBL were measured. The three endpoints were death, cardiovascular and cerebrovascular events (CCEs) and the composition of them. Univariate and multivariate Cox regression identified factors associated with the three endpoints respectively. X-tile analyses determined the optimal cut-off values for high risks. Restricted cubic spline plots illustrated the dose–response relationships. Correlations between the complement factors and risk factors for CCDs were analyzed.

**Results:**

Baseline plasma C4 was finally selected by univariate and multivariate Cox regression analyses for three endpoints, including all-cause mortality, CCEs and the composition of them. When baseline plasma C4 exceeded 0.47 (*P* = 0.001) or 0.44 (*P* = 0.018) g/L respectively, the risks for death or achieving the composite endpoint enhanced significantly. The relationships of C4 and HR for the three endpoints showed a positive linear trend. Plasma C4 had prominent correlations with blood TG (r = 0.62, *P* < 0.001) and HDL (r = -0.38, *P* < 0.001).

**Conclusions:**

A higher baseline plasma C4 level was significantly associated with the future incidence of decease, CCEs and either of them. Plasma C4 level correlated with blood TG and HDL.

**Supplementary Information:**

The online version contains supplementary material available at 10.1186/s12882-022-02829-0.

## Introduction

Hemodialysis (HD) has dominated the renal replacement therapy for decades among more than 2,000,000 patients afflicted with end-stage renal disease (ESRD) [1, 2]. Despite tremendous progress in HD techniques, the mortality and morbidity of complications (cardiovascular, cerebrovascular and infection diseases especially) remain extremely high [3]. Chronic kidney disease (CKD) is proved as an independent risk factor for all-cause mortality as well as cardiovascular and cerebrovascular diseases (CCDs) [4, 5]. Although maintenance HD contributes to extending the patients’ life against kidney failure, it also poses vascular injury on the already compromised cardio-cerebrovascular system [6, 7].

Recent decades have witnessed a series of studies about risk factors for mortality and complications in HD patients [8]. Apart from traditional risk factors (such as aging, comorbidities, obesity and dyslipidemia), more emerging risk factors (such as oxidative stress, endothelial dysfunction and chronic inflammation) [9] are identified to be substantially significant. Since the first report about the influence of HD on complement system [10], complement activation during HD has been thoroughly investigated [11–14]. Previously, our cross-sectional study also observed complement activation among 108 HD patients, representing a decreased level of plasma C3c and complement factor B (CFB), and an elevated level of plasma mannose-binding lectin (MBL), C3a and C5a, compared with normal controls [15]. Further, diverse complement proteins, including MBL [16–18], C3 [19], C1q-adiponectin [2], membrane attack complex (MAC) [19], complement factor H (CFH) [20] and complement receptor 1 (CR1) [21] were confirmed as significant predictors for the incidence of cardiovascular events or death for HD patients. Besides, a body of large studies targeting normal population also revealed plasma C3, C4 [22] and MBL [23, 24] level were risk factors for cardiovascular diseases.

In the present study, we took advantage of a prospective cohort to identify the pivotal components of complement system associated with the outcomes of HD patients. Other risk factors were also taken into consideration to control confounding variables. Determination of the optimal cut-off points for the identified factors is of considerable reference value for the prediction of the adverse events.

## Materials and methods

### Patients

A prospective study of 52 months (from October 2016 to February 2021) was conducted in a cohort of 108 patients on maintenance HD, recruited from a single center of Peking University First Hospital. All experiments were performed in accordance with relevant guidelines and regulations. The protocol has been described previously [15]. In brief, all patients were on a three times weekly dialysis schedule and spKt/V >  = 1.2. Patients with a significant inflammatory illness were excluded, defined as hypersensitive C-reactive protein (hs-CRP) > 50 mg/L.

### Data collection and follow-up

The severity of baseline comorbidities was assessed by the modified Charlson comorbidity index (mCCI), which was reported as a strong predictor for mortality in HD patients [25]. The mCCI is based on 19 certain comorbidities and excludes the subject’s age (Table S1) and can serve as a prediction tool for 10-year survival [25, 26].

During the study period, data on death and cardiovascular or cerebrovascular events (CCEs), considered as two endpoints respectively, were collected prospectively according to the medical records. Cardiovascular events were defined as the occurrence of ischemic heart disease [unstable angina pectoris, myocardial infarction, coronary artery bypass grafting (CABG) and/ or percutaneous coronary intervention (PCI)], sudden cardiac death and congestive heart failure (diagnosed according to the Modified Framingham clinical criteria) [17, 27]. Cerebrovascular events were defined as stroke (confirmed by neuroimaging), transient ischemic attack (TIA) (according to the AHA/ASA scientific statement of TIA in 2009) [28], or newly diagnosed > 70% stenosis of the extracranial carotid artery [17]. The composite endpoint, “decease or CCEs”, was also assessed. Besides, censoring events were considered as renal transplantation, transfers to other HD centers or achieving the end of the study, and the date was recorded as the final follow-up date.

### Clinical and laboratory measurements

Baseline clinical characteristics of the cohort have been published previously, as well as other laboratory measurements [15]. In summary, baseline demographic information was acquired from medical records, and baseline blood samples were obtained before the start of a regular 4-h HD session for regular laboratory tests and the quantification of complement components. Plasma C3a, C5a, MBL and MAC (sC5b-9) levels were detected by commercial ELISA kits from Quidel Corporation (San Diego, CA) [15]. We applied immunity transmission turbidity kits (Shanghai Beijia Biochemistry Reagents Co., Ltd) to quantify plasma C3c, CFB, CFH, C1q and C4 levels [15]. All the experiments were performed in accordance with the manufacturer’s instructions.

### Sample size

We calculated the sample size based on the previous reports stating the mortality rate of HD patients as around 0.15 [16, 29]. PASS software 15 (NCSS LLC., Kaysville, U.T., USA) was used for sample size calculation. Based on the statistical experience, a standard deviation as 1.50 of the log hazard ratio on a covariate was determined in the Cox regression. To achieve 80% power at a 0.05 significance level with two sides, a sample of 97 observations was recommended with a regression coefficient equal to 0.55. The sample size was adjusted since a multiple regression of the variable of interest on the other covariates in the Cox regression was expected to have an R-Squared of 0.20. Considering a 10% loss of follow-up rate, 108 observations were included in the study.

### Statistical analyses

Statistical evaluation was conducted with SPSS 25.0 (SPSS Inc., Chicago, IL, USA) and GraphPad Prism v.8 (La Jolla, CA, USA). Continuous parameters were presented as mean ± standard deviation or median (interquartile range), while categorical variables as proportions. In between-group comparisons were done using chi-square tests, t-test or Mann–Whitney U test. Survival analyses were performed by using the univariate and multivariate Cox regression for three endpoints respectively. Considering the event number [30], two confounders (with the lowest p value and reported important [31]) were applied to adjust in the multivariate Cox regression. We calculated the variance inflation factor (VIF) values and tolerance to evaluate collinearity between variables, with VIF > 10 and tolerance < 0.1 considered indicative of collinearity. False discovery rate (FDR) was applied to do multiple testing by using an FDR calculator (at http://www.rowett.ac.uk/Bgwh/fdr.html4). X-tile 3.6.1 software (Yale University, New Haven, CT, USA) [32] was exploited to determine the optimal cut-off value for plasma C4 level in our HD cohort. R 4.1.0 (The R Foundation, Vienna, Austria) was applied to visualize restricted cubic spline models (with 4 knots at 5th, 35th, 65th, 95th percentiles of C4 by RMS package) and correlation map (with Spearman’s correlation coefficients by corrplot package). *P* < 0.05 and FDR < 0.1 were considered statistically significant. All confidence intervals (CIs) were stated at a 95% confidence level.

## Results

### Patient characteristics and outcomes

A total of 108 patients with maintenance HD were enrolled according to the inclusion and exclusion criteria. The characteristics of the patient population have been reported previously [15]. In brief, there were 62 males and 46 females aged 56 ± 12, undergoing HD therapy for 60 (29,122) months at baseline. Other general characteristics probably correlated with prognosis and plasma complement factors (C3c, C1q, CFH, CFB, C4, MAC, C5a, C3a and MBL) are shown in Table 1, stratified by death or alive.

**Table 1:** Baseline characteristics of the HD cohort stratified by outcome

	All patients (*N* = 108)	Died (*N* = 17)	Alive ^a^(*N* = 91)	P
**Clinical characteristics**				
Age(years)	56(46,65)	64(52,77)	54(44,64)	0.010^*^
Gender(male/female)	62(57.4%) / 46(42.6%)	9(52.9%) / 8(47.1%)	53(58.2%) / 38(41.8%)	0.685
HD duration(months)	60(29,122)	74(34,155)	58(27,113)	0.299
Follow-up time(months)	52(39,52)	37(16,41)	52(52,52)	< 0.001^*^
mCCI	3(2,4)	5(2,5)	3(2,4)	0.060
SBP(mm Hg)	152 ± 22	142 ± 26	154 ± 20	0.037^*^
DBP(mm Hg)	77 ± 15	72 ± 18	79 ± 14	0.080
MAP(mm Hg)	101 ± 18	95 ± 19	104 ± 13	0.097
PP(mm Hg)	74 ± 21	70 ± 18	75 ± 21	0.367
Hemoglobin(g/L)	112.80(105.35,118.00)	107.39(102.55,119.95)	113.00(106.67,117.6)	0.236
WBC(× 10^9/L)	6.16(5.18,7.74)	6.87(4.33,8.18)	6.12(5.22,7.58)	0.565
PLT(× 10^9/L)	164.38 ± 53.48	131.77 ± 50.35	169.62 ± 52.36	0.013^*^
Glucose(mmol/L)	6.12(5.22,7.92)	6.87(4.33,8.18)	6.03(5.20,7.41)	0.176
Albumin(g/L)	40.75(38.35,42.45)	37.76 ± 3.86	40.683.52	0.003^*^
Hs-CRP(mg/L)	1.89(0.57,4.82)	4.42(2.28,10.40)	1.72(0.47,4.38)	0.045^*^
SF(ug/L)	296.99 ± 165.25	299.37 ± 140.31	296.59 ± 169.85	0.956
eGFR(ml/min⋅1.73m^2^)	15.26(12.61,18.31)	15.78(14.03,20.50)	15.16(12.57,17.80)	0.788
spKt/V	1.53 ± 0.29	1.43 ± 0.35	1.54 ± 0.28	0.168
Phosphate(mmol/L)	1.67(1.40,2.13)	1.71(1.55,2.01)	1.66(1.40,2.14)	0.745
Calcium(mmol/L)	2.33 ± 0.28	2.26 ± 0.23	2.35 ± 0.28	0.258
PTH(pg/ml)	328.89(169.80,487.40)	287.43(121.31,419.14)	348.46(172.24,525.92)	0.216
**Complement factors**				
C3c(g/L)	0.92 ± 0.17	0.98 ± 0.21	0.91 ± 0.16	0.154
C1q(mg/L)	201.84 ± 41.43	209.34 ± 39.79	200.44 ± 41.79	0.419
CFH(ug/mL)	361.77 ± 57.63	388.64 ± 75.32	361.21 ± 57.06	0.169
CFB(mg/L)	346.15(299.93,388.20)	355.5(288.55,430.70)	346.10(302.40,387.20)	0.358
C4(g/L)	0.31(0.25,0.38)	0.33(0.28,0.49)	0.31(0.24,0.38)	0.205
MAC (ng/mL)	482.26(307.59,783.75)	401.80(325.65,981.94)	484.32(294.83,758.05)	0.440
C5a(ng/mL)	31.03 ± 10.80	28.13 ± 9.70	31.57 ± 10.97	0.230
C3a(ng/mL)	238.72(190.12,318.95)	280.29(224.61,346.60)	229.03(182.10,294.49)	0.339
MBL(ng/mL)	4346.38(1415.73,8979.95)	4096.73(923.94,8756.41)	4807.28(1466.52,9043.82)	0.471
Primary cause of ESRD				0.415
**Primary glomerulopathy**	37(34.3%)	5(29.4%)	32(35.2%)	
Diabetes	14(13.0%)	2(11.8%)	12(13.2%)	
Hypertension	14(13.0%)	4(23.5%)	10(11.0%)	
ADTKD	10(9.3%)	0	10(11.0%)	
Tubulointerstitial nephropathy	17(15.7%)	2(11.8%)	15(16.5%)	
Other or unknown	16(14.8%)	4(23.5%)	12(13.2%)	
Comorbidity				
CCDs	39(36.1%)	9(52.9%)	30(33.0%)	0.116
Hypertension	77(71.3%)	8(47.1%)	69(75.8%)	0.034^*^
Diabetes	12(11.1%)	2(11.8%)	10(11.0%)	1.000

Data are shown as mean ± SD or median (interquartile range) for continuous variables and proportions for categorical variables. *P* < 0.05 are marked with ^*^

^a^ The alive refers to patients who weren’t dead until the end of their censoring time (*N* = 91), including those undergoing maintenance hemodialysis (*N* = 75) and receiving renal transplantation or transferring to other hospitals (*N* = 16) during the follow-up

HD duration, hemodialysis duration; *mCCI*, modified Charlson comorbidity index, *SBP* systolic blood pressure, *DBP* diastolic blood pressure, *MAP* mean arterial blood pressure, *PP* pulse pressure, *WBC* white blood cell, *PLT* blood platelet, *Hs-CRP* high-sensitivity C-reactive protein, *SF* serum ferritin, *eGFR* estimated glomerular filtration rate, *PTH* parathyroid hormone, *CFH* complement factor H, *CFB* complement factor B, *MAC* membrane attack complex, complement C5b-9; *MBL* mannose-binding lectin

**Fig. 1:**
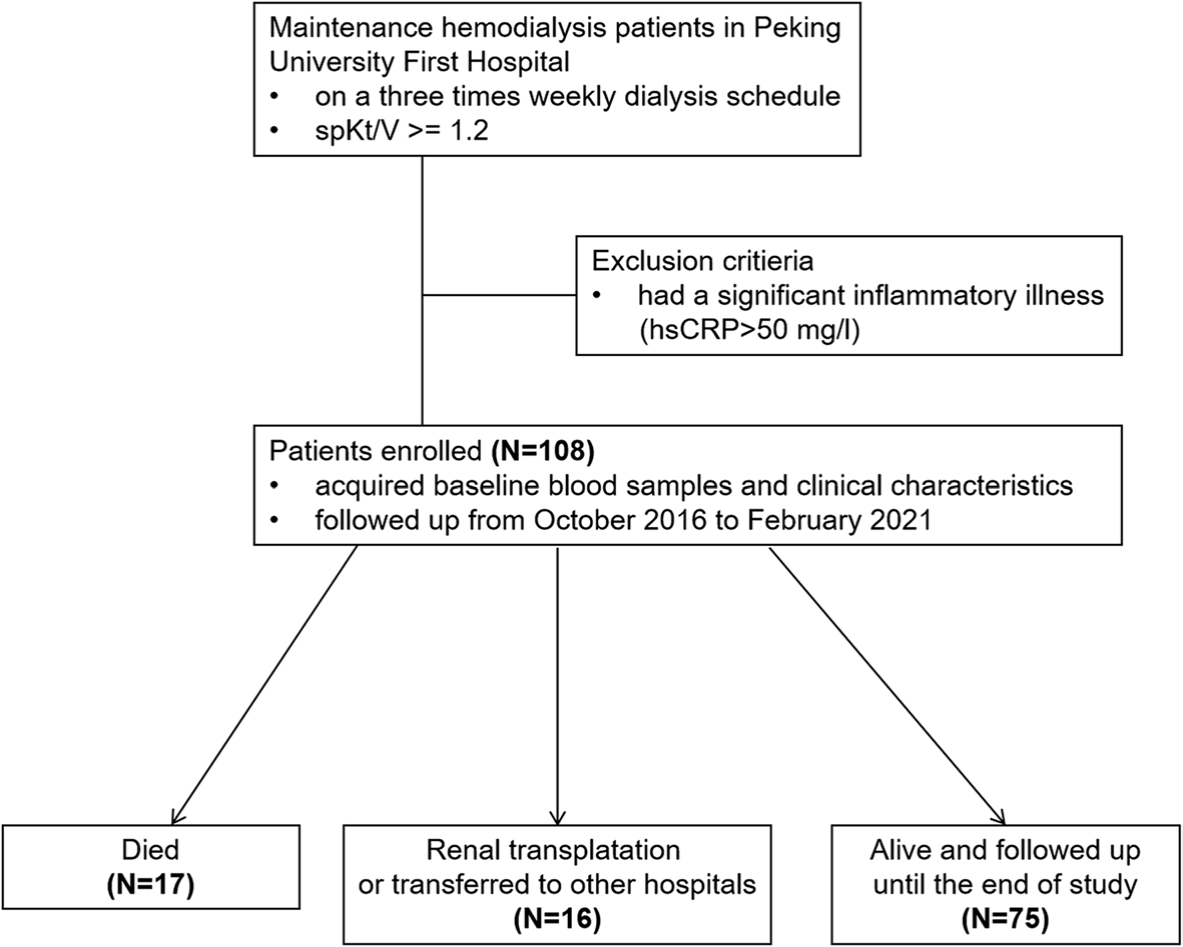
Flow diagram of study design, patient recruitment and outcomes

**Fig. 2:**
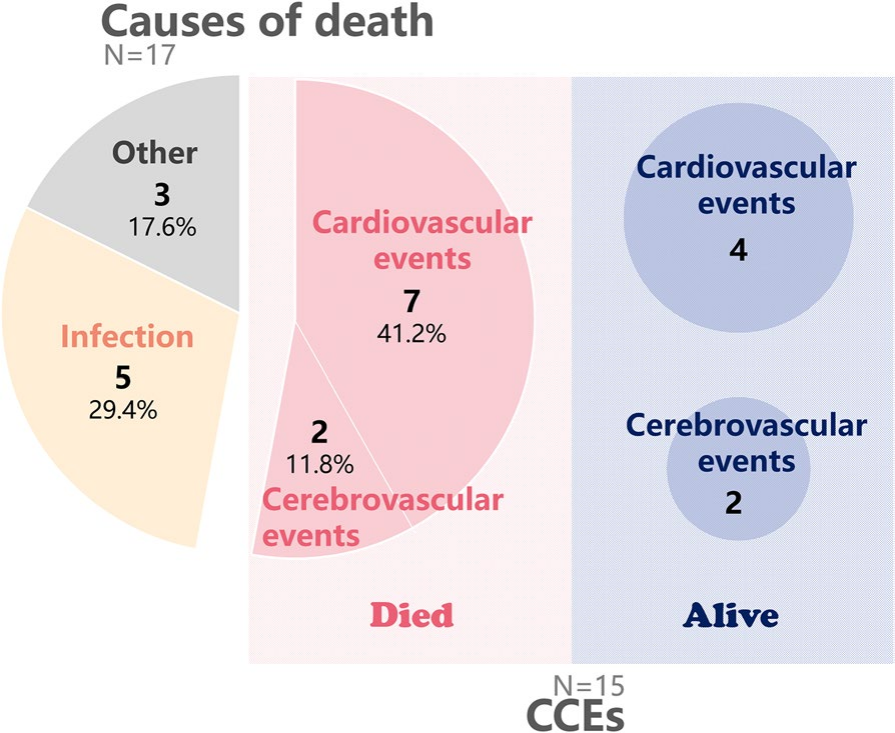
Causes of death and cardiovascular and cerebrovascular events (CCEs) in HD patients. Among the 108 HD patients, 17 deaths and 15 CCEs were recorded during the follow-up time. Cardiovascular and cerebrovascular events were the primary cause of death, accounting for 41.2% as seven and 11.8% as two respectively. Five patients died of infection, and three of the other causes including cancer, gastrointestinal hemorrhage and acute pancreatitis. In those 15 cases attacked by CCEs, six survived and were alive until the end of follow-up. In total, 23 patients achieved the composite endpoint

During 52 months follow-up period, 17 patients died. Sixteen survived patients received renal transplantation or were transferred to other dialysis centers, and the other 75 maintained HD in our center (Fig. 1). The primary cause of death of the 17 patients was cardiovascular (*n* = 7, 41.2%) and cerebrovascular (*n* = 2, 11.8%) events (CCEs). Infection (*n* = 5, 29.4%) took up the secondary place. Moreover, other six cases attacked by cardiovascular (*n* = 4) or cerebrovascular (*n* = 2) events, survived and maintained HD until the end of the study. In total, 23 patients achieved the composite endpoint (Fig. 2).

### Clinical parameters and plasma complement factors associated with the prognosis by univariate Cox regression analysis

To identify the correlation between clinical parameters and the prognosis of HD patients, we collected multiple possible indexes reported previously [8] to conduct univariate Cox regression for three endpoints, including death, CCEs and the composite endpoint (Table 2). Seven factors have been identified to be significantly associated with all-cause mortality, one with the incidence of CCEs and four with the composite endpoint. Remarkably, a high level of plasma C4 was significantly associated with all of the three endpoints (HR, 5.039; 95%CI, 1.337–18.998; *P* = 0.017 for all-cause mortality, HR, 4.497; 95%CI, 1.117–18.104; *P* = 0.034 for CCEs, HR, 3.927; 95%CI, 1.120–13.769; *P* = 0.033 for the composite endpoint). Besides, only the FDR of C4 for the all-cause mortality was calculated < 0.1 (equal to 0.051) among 9 complement factors. To further visualize the distribution of plasma C4 levels, histograms were plotted (Figure S1). Aging was associated both with an increased all-cause mortality (HR, 1.059; 95%CI, 1.012–1.108; *P* = 0.013) and incidence of composite endpoint (HR, 1.054; 95%CI, 1.015–1.094; *P* = 0.006), and mCCI score showed a similar trend (HR, 1.295; 95%CI, 1.034–1.622; *P* = 0.024 for all-cause mortality, HR, 1.245; 95%CI, 1.025–1.512; *P* = 0.027 for the composite endpoint). An elevated blood platelet count was associated both with reduced risk of all-cause mortality (HR, 0.987; 95%CI, 0.978–0.997; *P* = 0.009) and incidence of the composite endpoint (HR, 0.991; 95%CI, 0.983–0.999; *P* = 0.032).

**Table 2:**
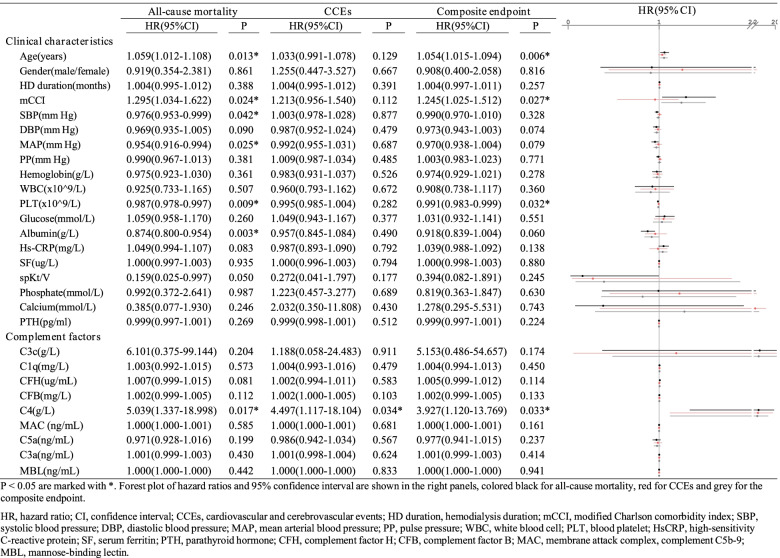
Univariate Cox regression analyses for 3 endpoints

*P* < 0.05 are marked with ^*^. Forest plot of hazard ratios and 95% confidence interval are shown in the right panels, colored black for all-cause mortality, red for CCEs and grey for the composite endpoint. Only the FDR of C4 for the all-cause mortality was calculated < 0.1 (equal to 0.051) among 9 complement factors

*HR* hazard ratio, *CI* confidence interval, *CCEs* cardiovascular and cerebrovascular events, *HD duration* hemodialysis duration, *mCCI* modified Charlson comorbidity index, *SBP* systolic blood pressure, *DBP* diastolic blood pressure, *MAP* mean arterial blood pressure, *PP* pulse pressure, *WBC* white blood cell, *PLT* blood platelet, *HsCRP* high-sensitivity C-reactive protein, *SF* serum ferritin, *PTH* parathyroid hormone, *CFH* complement factor H, *CFB* complement factor B, *MAC* membrane attack complex, complement C5b-9, *MBL* mannose-binding lectin, *FDR* false discovery rate

### Clinical parameters and plasma complement factors associated with the prognosis by multivariate Cox regression analysis

For further exploration, we then performed multivariate Cox regression analyses to control confounding variables (Table 3). Model I was constructed to adjust for age and PLT because of their low p values and clinical importance reported before [31]. Furthermore, there was no collinearity among the independent variables as their VIF < 10 and tolerance > 0.1 (Table S3).

**Table 3:** Multivariate Cox regression analyses for 3 endpoints

	All-cause mortality		CCEs		Composite endpoint	
	Non-adjusted	Model I	Non-adjusted	Model I	Non-adjusted	Model I
		*P* < 0.001		*P* = 0.008		*P* < 0.001
C4(g/L)	5.04 (1.34–19.00)	46.70 (6.80–320.67)	4.50 (1.12–18.10)	6.40 (1.49–27.44)	3.93 (1.12–13.77)	14.66 (3.00–71.69)
	*P* = 0.017	*P* < 0.001	*P* = 0.034	*P* = 0.013	*P* = 0.033	*P* = 0.001
Age(years)	1.06 (1.01–1.11)	1.09 (1.03–1.15)	1.03 (0.99–1.08)	1.05 (1.00–1.10)	1.05 (1.02–1.09)	1.07 (1.02–1.11)
	*P* = 0.013	*P* = 0.003	*P* = 0.129	*P* = 0.048	*P* = 0.006	*P* = 0.003
PLT(× 10^9/L)	0.99 (0.98–0.99)	0.98 (0.97–0.99)	1.00 (0.99–1.00)		0.99 (0.98–0.99)	0.99 (0.98–0.99)
	*P* = 0.009	*P* = 0.002	*P* = 0.282	*P* = 0.167	*P* = 0.032	*P* = 0.015

According to the model above, increased plasma C4 showed a significant association with the incidence of all three adverse endpoints, while blood platelet count showed the opposite effect. Specifically, a higher baseline plasma C4 was associated with a worse prognosis, including an increased risk of death (HR, 46.70; 95%CI, 6.80–320.67; *P* < 0.001 in model I), incidence of CCEs (HR, 6.40; 95%CI, 1.49–27.44; *P* = 0.013 in model I) or achieving the composite endpoint (HR, 14.66; 95%CI, 3.00–71.69; *P* = 0.001 in model I). The patients with higher blood platelet count seemed to have reduced risk of death (HR, 0.98; 95%CI, 0.97–0.99; *P* = 0.002 in model I) or achieving the composite endpoint (HR, 0.99; 95%CI, 0.98–0.99; *P* = 0.015 in model I).

### Outcome-based cut-point optimization of complement factor 4 by X-tile analysis

**Fig. 3:**
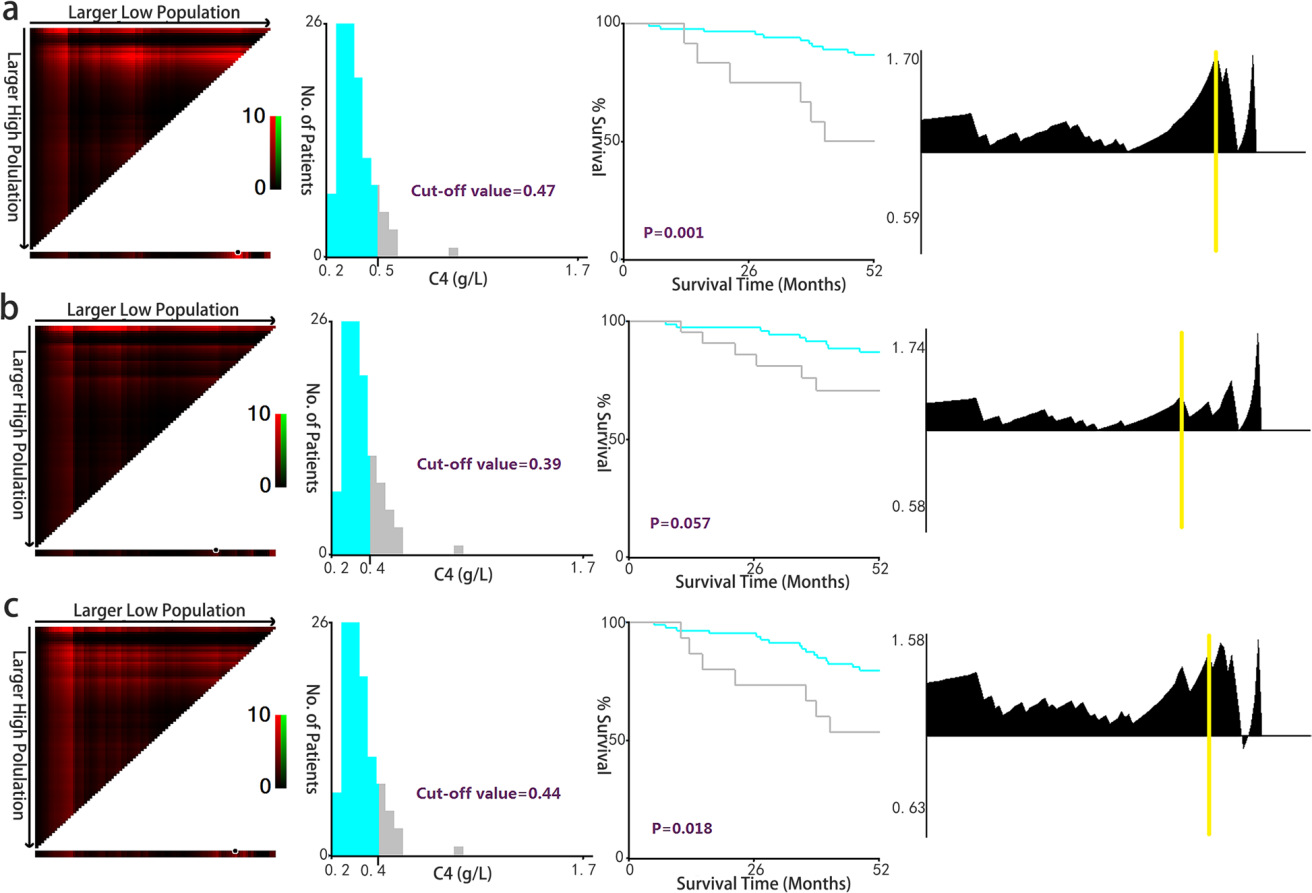
X-tile analyses: determination of optimal cut-off values of plasma C4 for 3 endpoints. X-tile plots of 108 HD patients are shown in the first panels. The optimal cut-off values highlighted by the black circles in the first panels are detailed in the second panels, which are histograms revealing a continuous distribution based on plasma C4. Kaplan–Meier plots are displayed in third panels, with *P* values of the corresponding optimal cut-off value. The fourth panels demonstrate relative risk plots and the cut-off points are marked by yellow vertical bars. **a** The optimal cut-off value for all-cause death was 0.47 (X^2^ = 11.386, *P* = 0.001). **b** The optimal cut-off value for CCEs was 0.39 (X^2^ = 3.615, *P* = 0.057). **c** The optimal cut-off value for the composite endpoint was 0.44 (X^2^ = 5.616, *P* = 0.018)

Since the baseline plasma C4 level may predict the prognosis of the cohort, X-tile analyses were performed (Fig. 3). We tried to determine the optimal cut-off values for plasma C4 to identify patients with a high risk for adverse outcomes. X-tile plots of the HD cohort displayed the optimal cut-off values. Histogram analyses of plasma C4 level showed a continuous distribution and were separated by the values in two colors. These divisions were applied to chart Kaplan–Meier plots and calculate the corresponding Log Rank (Mantel-Cox) chi-square and *P* values. In total, X-tile analyses revealed that once plasma C4 was higher than 0.47 (X^2^ = 11.386, *P* = 0.001) or 0.44 (X^2^ = 5.616, *P* = 0.018) g/L respectively, the risk of death (Fig. 3a) or suffering either death or being attacked by CCEs (Fig. 3c) increased significantly. The optimal cut-off value for CCEs was 0.39 g/L (Fig. 3b), but with no statistical significance (X^2^ = 3.615, *P* = 0.057).

### Dose-response analysis of plasma C4 level with prognosis by restricted cubic spline model

**Fig. 4:**
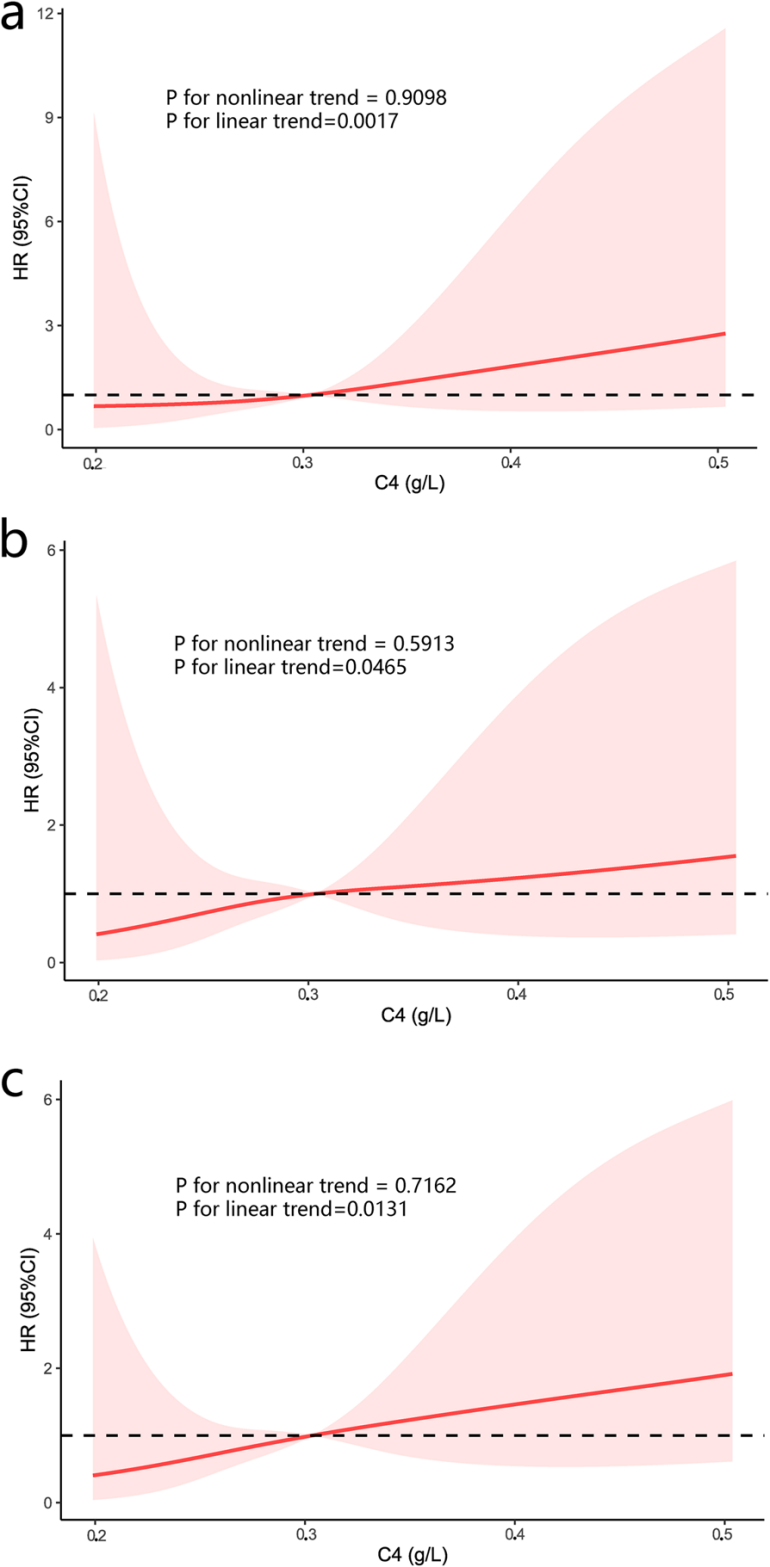
Association of plasma C4 level with the 3 endpoints in a restricted cubic spline model. Analyses were adjusted for age and PLT with 4 knots at 5th, 35th, 65th, 95th percentiles of C4. Multivariable adjusted hazard ratios (HRs; red line) with 95% CI (pink area) demonstrated the linear association of plasma C4 level with all-cause mortality **a**, CCEs **b** and the composite endpoint **c**

A restricted cubic spline model with 4 knots at 5th, 35th, 65th, 95th percentiles of C4 (Fig. 4) was employed to simulate the relationship between plasma C4 level and the risk for three endpoints. The model was adjusted with age and PLT. The relationships between plasma C4 level and HR for death (P for nonlinear trend = 0.9098, for linear trend = 0.0017, Fig. 4a), the incidence of CCEs (P for nonlinear trend = 0.5913, for linear trend = 0.0465, Fig. 4b) and the composite endpoint (P for nonlinear trend = 0.7162, for linear trend = 0.0131, Fig. 4c) were all observed as a linear tendency.

### Correlations between complement factors and the traditional risk factors for CCDs at baseline

To further investigate possible mechanisms for the relationship between complement C4 and the prognosis, we assessed the correlations between complement factors and the traditional risk factors for CCDs at baseline. Apart from age, gender, blood pressure and diabetes mellitus or not, we detected the baseline blood lipids, containing triglyceride (TG), total cholesterol (TC), low-density lipoprotein cholesterol (LDL) and high-density lipoprotein cholesterol (HDL) among 78 of the 108 HD patients. No significant difference in baseline characteristics between the 78 patients and the whole (Table S2).

**Fig. 5:**
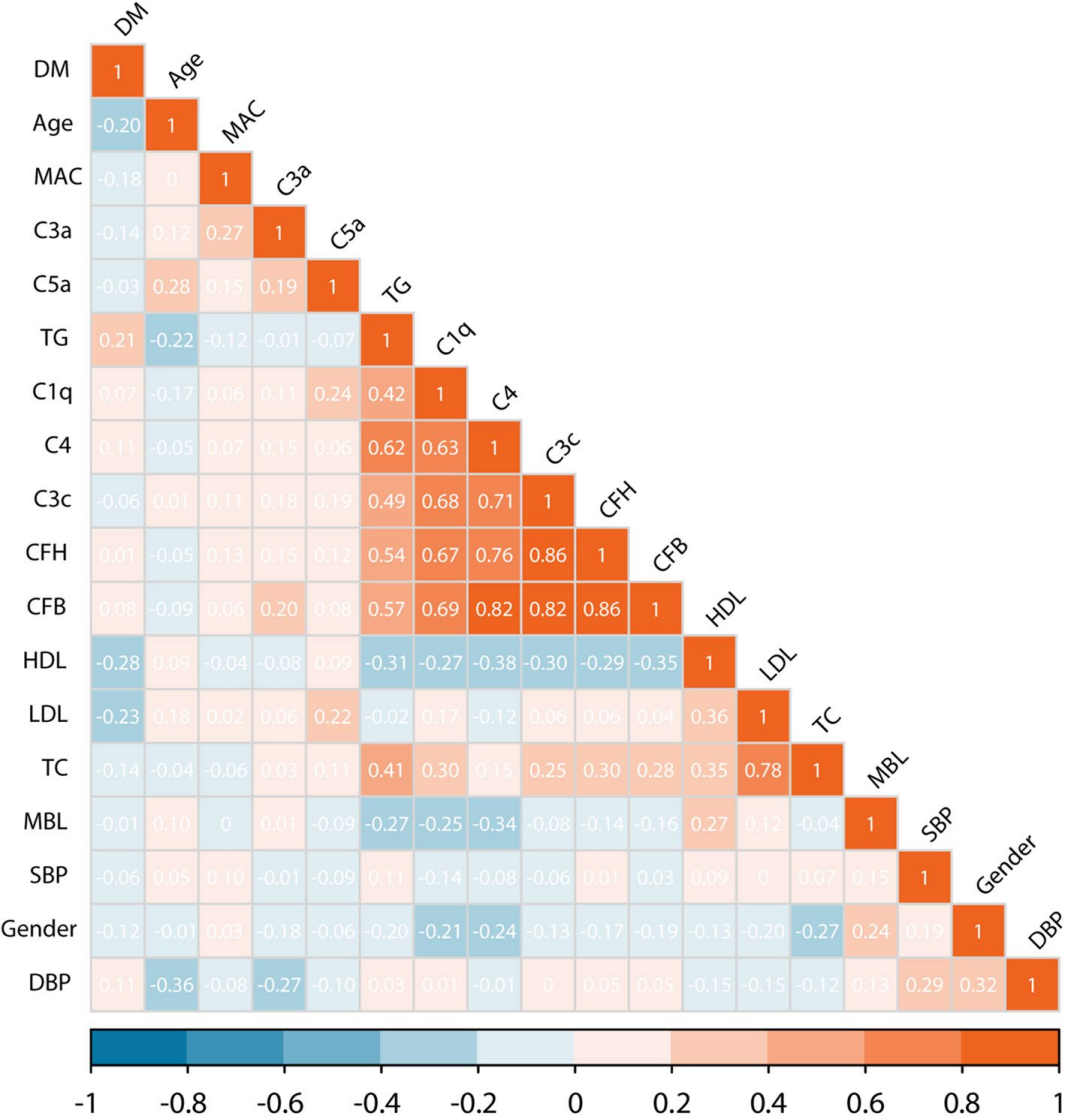
Correlation diagram of the complement factors and the traditional risk factors for CCEs. Spearman’s correlation coefficients between the variables are shown as numbers with the corresponding grids colored according to the values. *DM* diabetes mellitus, *MAC* membrane attack complex, complement C5b-9, *TG* triglyceride, *CFH* complement factor H, *CFB* complement factor B, *HDL* high-density lipoprotein cholesterol, *LDL* low-density lipoprotein cholesterol, *TC* total cholesterol, *MBL* mannose-binding lectin, *SBP* systolic blood pressure, *DBP* diastolic blood pressure

Spearman’s correlation analyses (Fig. 5) indicated the strong positive correlations between C4, CFB, CFH and C3c, especially between CFB and C4 (r = 0.82, *P* < 0.001), CFH (r = 0.86, *P* < 0.001) and C3c (r = 0.82, *P* < 0.001). Moreover, C4 exhibited a prominent correlation with blood lipids, primarily with TG (r = 0.62, *P* < 0.001) and HDL (r = -0.38, *P* < 0.001) (Figure S2). Conversely, in our HD cohort, no significant correlations were revealed between C4 and age (r = -0.05, *P* = 0.941), SBP (r = -0.08, *P* = 0.069), DBP (r = -0.01, *P* = 0.956) and diabetes mellitus (r = 0.11, *P* = 0.668).

## Discussion

The present study showed an association between the baseline plasma C4 level and the adverse outcomes, including all-cause mortality and CCEs, among patients receiving maintenance HD. Both in the unadjusted and adjusted models, plasma C4 level substantially showed a predictive value. Patients whose baseline plasma C4 > 0.47 g/L or 0.44 g/L in our cohort exhibited higher all-cause mortality or incidence of CCEs. Meanwhile, the level of plasma C4 manifested a positive linear trend with HR for death, CCEs and either of them. In our cohort, baseline plasma C4 levels had correlations with blood lipids, which were widely acknowledged as risk factors for the development of cardiovascular diseases. These findings suggested that C4 may participate in the pathological processes in patients with maintenance HD and excess plasma C4 predicted a worse prognosis for HD patients.

A body of evidence indicated that multiple complement components related to outcomes of HD patients, covering MBL [16–18], C3 [19], C1q-adiponectin [2], MAC [19], CFH [20] and CR1 [21]. A higher level of plasma C3 before an HD session, was reported to be associated with a higher probability of cardiovascular events [19]. Baseline sC5b-9 levels was predicted to be correlated with cardiovascular events and mortality. A lower level of serum C1q-adiponectin/C1q ratios were also identified as a prognostic marker of cardiovascular diseases [2]. Thus, a possible explanation would be that both an elevated complement activation and an intensified complement activity have been the risk factors for cardiovascular diseases. Additionally, Satomura et al. revealed that a lower MBL level could independently predict all-cause mortality in HD patients [16], which was also proposed to be linked with the morbidity of cardiovascular diseases in HD patients [17] and linked to accelerating arterial stiffness in HD patients [33].

Significantly, the diversity of the above conclusions mainly resulted from the heterogeneity of the patients. The patients’ characteristics varied among different HD centers, particularly such as, age, ethnicity, HD duration, the primary cause of ESRD and comorbidities. These differences profoundly influenced the distribution of the plasma complement levels among patients. Specifically, patients in Satomura’s study (9.054 ± 5.115 μg/ml) had a higher level of MBL than ours [4.346(1.415, 8.979)μg/ml] likely because of heterogeneity. Although a lower level of MBL wasn’t regarded as a significant risk factor in the current study, we indeed found a slight tendency likewise in our cohort. There was a considerable amount of the death whose MBL level was lower than 2.5 μg/ml [as 7 of 17 patients (41.2%)] (Figure S3). According to the above, conclusions in this field should be restricted to the certain population for higher accuracy. Furthermore, HD patients with any suspicious risk values should be carefully monitored and cared to decrease mortality and the incidence of CCEs.

Our previous cross-sectional study in the same cohort, found that the complement system was activated in patients on hemodialysis and a higher plasma C3a level prior a dialysis session was associated with severe abdominal aortic calcification [15]. Thus, we included all measured plasma complement factors to identify the critical components associated with the outcomes. As a consequence, an elevated level of plasma C4 was proposed to be the risk factor that significantly increased all-cause mortality and incidence of CCEs, independent of other risk factors reported previously [8]. Although plasma C4 level among hemodialysis patients (0.312 g/L (0.25 g/L,0.38 g/L)) wasn’t prominently higher (*P* = 0.10) than the normal (0.285 g/L (0.22 g/L,0.39 g/L)) [15], an increased level of plasma C4 could discriminate patients with adverse outcomes.

In the complement cascades, C4 contributes to the formation of C3 convertase in the classical and lectin pathway. Circulating C4 and C3 mainly derive from hepatocytes [34] and are also related to adipose tissue variables [35] and involved in the development of visceral adiposity [36]. In healthy individuals, the polymorphism of C4 genes, including the variation of the gene copy number, the gene size and the C4 isotypes (C4A and C4B), largely determines the plasma levels and functions of C4 [37]. An elevated level of plasma C4, as well as C3, are reported as strong inflammatory indicators of metabolic syndrome [38, 39], cardiovascular diseases [22], thrombotic diseases [40] and allergic diseases [41]. During the pathological process, C4 and C4a may play pivotal roles in chronic inflammation and tissue injury, rather than defending against pathogens and cleaning immune complex and cells [42]. The elevated systemic C4 and C3 levels were probably correlated with metabolic syndrome [38, 39, 42], which is proved to raise the risk of cardiovascular disease, diabetes and all-cause mortality among general population [43]. Although our study confirmed the critical impact of plasma C4, plasma C3 level wasn’t measured in the analyses. As we mentioned above, in those studies reported the correlation between C3 and prognosis, the plasma C4 levels were not measured. Further investigations are also needed to answer the relationship between C3 and C4, and their predictive value for prognosis in patients with maintenance HD.

Apart from the well-known conjunction in complement pathways, C4 may have distinct effects on metabolism and chronic inflammation [38]. Studies of human populations have shown that C3 and C4 are associated with the incidence of myocardial infarction and stroke [22], as well as with their risk factors, such as obesity, hypertension, hyperlipemia and diabetes [28–31]. Analogous associations were sighted in our HD cohort between plasma C4 and the incidence of CCEs and hyperlipemia. Cytokines stimulating the hepatic production of C4 may also induce hyperlipemia and undermine insulin sensitivity. C4 binding protein (C4BP) inhibits the classical and lectin pathway by binding to C4b and is reported as a protective factor for desired blood pressure, fasting blood glucose and cell function [44, 45]. Furthermore, C4a, the product of C4 activation, may participate in cardiac remodeling and inflammation [46] by binding to protease-activated receptor I (PAR1) [47]. Other components participating in the C4 activation, for example, platelets and endothelial cells [38], are receiving increasing attention due to their crosstalk in inflammation and vascular injury [48, 49]. In our study, plasma C4 showed strong correlations with CFH, CFB, C1q and C3c. Thus, the impact of C4 on the prognosis in HD patients is likely to exert through the classic and alternative pathways.

Other baseline variables in our study, including demographic characteristics and laboratory measurements, were also included in the analyses as confounding factors. According to the univariate Cox regression, age, albumin, blood pressure, mCCI and comorbidity conditions were associated with prognosis, in consistent with previous studies [8, 25, 50–55]. Besides, a low level of blood platelet count was identified as a risk factor in our study. However, previous studies revealed that those with a high platelet count (> 300 × 10^9/L) exhibited higher cardiovascular mortality [56]. Given the fact above, therapies targeting or affecting platelet need to be individualized and refined among HD patients. The dialyzer is considered to exert remarkable impacts on the count, morphology and function of platelet [57, 58], worsening the already undesired platelet dysfunction (thrombosis and bleeding diathesis) in patients with ESRD [56]. Considerable activation of platelet can occur during HD session, owing to the exposure to dialysis membrane [57]. Whether the platelet activation by dialyzer contributes to the elevated all-cause mortality and incidence of CCEs in HD patients remains inconclusive.

Nevertheless, there are some limitations in our study. The case volume of the prospective analyses was relatively low, so limited endpoint events were observed. These defects might weaken the power of tests, especially the multivariate Cox regression. Owing to the potential possibility of overfitting, the multivariate Cox regression models could only reveal the significance of plasma C4 levels rather than be applied as the predict tools for HD patients. Patients enrolled in our study were used as the training population to determine the hazard thresholds of plasma C4 level, thus a validation population is needed to further confirm the optimal value. C4 is cleaved by C1s [59] and mannan-binding lectin-associated serine protease 2(MASP2) [60] to release C4a and C4b to produce C3 convertase subsequently. Thus, whether the downstream fragments of C4 activation, such as C4a and C4d, have the correlations with the prognosis in HD patients is further to be excavated.

In conclusion, a high level of baseline plasma C4 was confirmed to be associated with all-cause mortality and the incidence of CCEs. Consequently, plasma C4 level is recommended as an innovative clinical predictor for HD patients, together with other risk-related variables, such as age, blood pressure, albumin, blood platelet count, etc. Further studies are required to thoroughly elucidate the significance and mechanisms of plasma C4 in HD patients.

## Supplementary Information

**Additional file 1:** **Figure S1. **Histograms of plasma C4 levels among the HD patients. **a.** The histogram of plasma C4 levels among the whole cohort. **b.** The histogram of plasma C4 levels in the died and the alive groups. **Figure S2. **Correlations between plasma levels of C4 and TG and HDL. **a. **Scatter plot for C4 and TG. Spearman’s rho=0.62, *p*<0.001. **b.** Scatter plot for C4 and HDL. Spearman’s rho=-0.38, *p*<0.001.TG: triglyceride; HDL: high-density lipoprotein cholesterol. **Figure S3. **The histogram of plasma MBL levels in the died and the alive groups. There was a considerable amount of the death whose MBL level was lower than 2.5μg/ml [as 7 of 17 patients (41.2%)]MBL, mannose-binding lectin.**Table S1. **Description of modified Charlson comorbidity index (mCCI) [23]. **Table S2. **Baseline characteristics of the HD cohort and patients with lipid tests. **Table S3. **The VIF and tolerance of the confounders in multivariate COX regression

## Data Availability

The datasets generated and/or analyzed during the current study are not publicly available due to the fact that the patients consented with their clinical statistics being used only for research but not shared to the public repository, but are available from the corresponding author on reasonable request.

## Abbreviations

HD: Hemodialysis
CCDs: Cardiovascular and cerebrovascular diseases
C3c: Complement factor 3c
C1q: Complement factor 1q
CFH: Complement factor H
CFB: Complement factor B
C4: Complement factor 4
MAC: Membrane attack complex
C5a: Complement factor 5a
C3a: Complement factor 3a
MBL: Mannose-binding lectin
CCEs: Cardiovascular and cerebrovascular events
ESRD: End-stage renal disease
CKD: Chronic kidney disease
CR1: Complement receptor 1
Hs-CRP: Hypersensitive C-reactive protein
mCCI: Modified Charlson comorbidity index
CABG: Coronary artery bypass grafting
PCI: Percutaneous coronary intervention
TIA: Transient ischemic attack
VIF: Variance inflation factor
FDR: False discovery rate
CIs: Confidence intervals
HR: Hazard ratio
eGFR: Estimated glomerular filtration rate
SBP: Systolic blood pressure
DBP: Diastolic blood pressure
MAP: Mean arterial blood pressure
PP: Pulse pressure
WBC: White blood cell
PLT: Blood platelet
SF: Serum ferritin
PTH: Parathyroid hormone
TG: Triglyceride
TC: Total cholesterol
LDL: Low-density lipoprotein cholesterol
HDL: High-density lipoprotein cholesterol
MetS: Metabolic syndrome
C4BP: C4 binding protein
PAR1: Protease-activated receptor I
MASP2: Mannan-binding lectin-associated serine protease 2
