# Supplementary material for: Plasma C4 level was associated with mortality, cardiovascular and cerebrovascular complications in hemodialysis patients
Source: BMC Nephrol. 2022 Jun 29;23:232. doi: 10.1186/s12882-022-02829-0 (PMC9245318; doi:10.1186/s12882-022-02829-0)
Supplement: Supplementary file 1 — Additional file 1: Figure S1. Histograms of plasma C4 levels among the HD patients. a. The histogram of plasma C4 levels among the whole cohort. b. The histogram of plasma C4 levels in the died and the alive groups. Figure S2. Correlations between plasma levels of C4 and TG and HDL. a. Scatter plot for C4 and TG. Spearman’s rho=0.62, p<0.001. b. Scatter plot for C4 and HDL. Spearman’s rho=-0.38, p<0.001.TG: triglyceride; HDL: high-density lipoprotein cholesterol. Figure S3. The histogram of plasma MBL levels in the died and the alive groups. There was a considerable amount of the death whose MBL level was lower than 2.5μg/ml [as 7 of 17 patients (41.2%)]MBL, mannose-binding lectin.Table S1. Description of modified Charlson comorbidity index (mCCI) [23]. Table S2. Baseline characteristics of the HD cohort and patients with lipid tests. Table S3. The VIF and tolerance of the confounders in multivariate COX regression [file 12882_2022_2829_MOESM1_ESM.docx]

Supplementary Material

# Supplementary Tables

**Table S1. Description of modified Charlson comorbidity index (mCCI) ^[23]^.**

| **Points** | **Variable** |  |
| --- | --- | --- |
| 1 | **Myocardial infarction**  History of definite or probable MI (EKG changes and/or enzyme changes) | |
|  | **Congestive heart failure**  Exertional or paroxysmal nocturnal dyspnea and has responded to digitalis, diuretics, or afterload reducing agents | |
|  | **Peripheral vascular disease**  Intermittent claudication or past bypass for chronic arterial insufficiency, history of gangrene or acute arterial insufficiency, or untreated thoracic or abdominal aneurysm (≥6 cm) | |
|  | **Cerebrovascular accident or transient ischemic attack**  History of a cerebrovascular accident with minor or no residua and transient ischemic attacks | |
|  | **Dementia**  Chronic cognitive deficit | |
|  | **Chronic obstructive pulmonary disease** | |
|  | **Connective tissue disease** | |
|  | **Peptic ulcer disease**  Any history of treatment for ulcer disease or history of ulcer bleeding | |
|  | **Mild liver disease**  Mild = chronic hepatitis (or cirrhosis without portal hypertension) | |
|  | **Uncomplicated diabetes** | |
| 2 | **Hemiplegia** | |
|  | **Moderate to severe chronic kidney disease**  Severe = on dialysis, status post kidney transplant, uremia, moderate = creatinine >3 mg/dL (0.27 mmol/L) | |
|  | **Diabetes with end-organ damage** | |
|  | **Localized solid tumor** | |
|  | **Leukemia** | |
|  | **Lymphoma** | |
| 3 | **Moderate to severe liver disease**  Severe = cirrhosis and portal hypertension with variceal bleeding history, moderate = cirrhosis and portal hypertension but no variceal bleeding history | |
| 6 | **Metastatic solid tumor** |  |
|  | **AIDS** |  |

**mCCI score =** $\sum\mathbf{points}$**.**

**Table S2. Baseline characteristics of the HD cohort and patients with lipid tests.**

|  | | **All patients**  **（N=108）** | | **Patients with lipid tests**  **（N=78）** | **P** |
| --- | --- | --- | --- | --- | --- |
| **Clinical characteristics** | | | |  |  |
|  | Age(years) | | 56±12 | 57±13 | 0.499 |
|  | Gender(male/female) | | 62(57.4%) / 46(42.6%) | 38(48.7%) / 40(51.3%) | 0.241 |
|  | HD duration(months) | | 60(29,122) | 67(26,151) | 0.515 |
|  | Follow-up time(months) | | 52(39,52) | 52(40,52) | 0.846 |
|  | mCCI | | 3(2,4) | 3(2,4) | 0.757 |
|  | SBP(mm Hg) | | 152±22 | 152±22 | 0.988 |
|  | DBP(mm Hg) | | 77±15 | 76±14 | 0.448 |
|  | MAP(mm Hg) | | 101(93,113) | 99(92,110) | 0.165 |
|  | PP(mm Hg) | | 74±21 | 74±24 | 0.944 |
|  | Hemoglobin(g/L) | | 112.69±10.49 | 112.16±9.78 | 0.734 |
|  | WBC(x10^9/L) | | 6.16(5.18,7.74) | 6.24(5.11,7.87) | 0.835 |
|  | PLT(x10^9/L) | | 164.38±53.48 | 163.90±52.06 | 0.953 |
|  | Glucose(mmol/L) | | 6.12(5.22,7.92) | 6.16(5.26,7.98) | 0.733 |
|  | Albumin(g/L) | | 40.75(38.25,42.45) | 40.80(38.45,42.35) | 0.968 |
|  | Hs-CRP(mg/L) | | 1.89(0.57,4.82) | 1.81(0.50,4.48) | 0.461 |
|  | SF(ug/L) | | 296.99±165.25 | 267.82±171.57 | 0.273 |
|  | eGFR(ml/min⋅1.73m2) | | 15.26(12.61,18.31) | 15.44(12.57,17.80) | 0.889 |
|  | spKt/V | | 1.53±0.29 | 1.54±0.29 | 0.704 |
|  | Phosphate(mmol/L) | | 1.77±0.52 | 1.77±0.53 | 0.926 |
|  | Calcium(mmol/L) | | 2.33±0.28 | 2.36±0.29 | 0.453 |
|  | PTH(pg/ml) | | 328.89(169.80,487.40) | 322.91(158.94,456.75) | 0.514 |
| **Complement factors** | | | | |  |
|  | C3c(g/L) | | 0.92±0.17 | 0.94±0.16 | 0.533 |
|  | C1q(mg/L) | | 201.84±41.43 | 205.48±39.01 | 0.546 |
|  | CFH(ug/mL) | | 361.77±57.63 | 370.30±60.75 | 0.598 |
|  | CFB(mg/L) | | 346.15(299.93,388.20) | 352.75(311.13,381.65) | 0.753 |
|  | C4(g/L) | | 0.31(0.25,0.38) | 0.31(0.26,0.37) | 0.932 |
|  | MAC (ng/mL) | | 482.26(307.59,783.75) | 483.20(307.15,783.10) | 0.830 |
|  | C5a(ng/mL) | | 31.03±10.80 | 31.13±10.62 | 0.950 |
|  | C3a(ng/mL) | | 238.72(190.12,318.95) | 228.42(186.11,318.20) | 0.769 |
|  | MBL(ng/mL) | | 4346.38(1415.73,8979.95) | 4682.83(1439.51,9054.55) | 0.832 |
| **Primary cause of ESRD** | | | | | 0.999 |
|  | Primary glomerulopathy | | 37(34.3%) | 27(34.6%) |  |
|  | Diabetes | | 14(13.0%) | 10(12.8%) |  |
|  | Hypertension | | 14(13.0%) | 9(11.5%) |  |
|  | ADTKD | | 10(9.3%) | 7(9.0%) |  |
|  | Tubulointerstitial nephropathy | | 17(15.7%) | 12(15.4%) |  |
|  | Other or unknown | | 16(14.8%) | 13(16.7%) |  |
| **Comorbidity** | | | | | |
|  | CCDs | | 39(36.1%) | 30(38.5%) | 0.743 |
|  | Hypertension | | 77(71.3%) | 55(70.5%) | 0.908 |
|  | Diabetes | | 12(11.1%) | 10(12.8%) | 0.722 |

Data are shown as mean ± SD or median (interquartile range) for continuous variables and proportions for categorical variables. P < 0.05 are bolded.

HD duration, hemodialysis duration; mCCI, modified Charlson comorbidity index; SBP, systolic blood pressure; DBP, diastolic blood pressure; MAP, mean arterial blood pressure; PP, pulse pressure; WBC, white blood cell; PLT, blood platelet; Hs-CRP, high-sensitivity C-reactive protein; SF, serum ferritin; eGFR, estimated glomerular filtration rate; PTH, parathyroid hormone; CFH, complement factor H; CFB, complement factor B; MAC, membrane attack complex, complement C5b-9; MBL, mannose-binding lectin.

**Table S3.** The VIF and tolerance of the confounders in multivariate COX regression**.**

|  | VIF | Tolerance |
| --- | --- | --- |
| Age | 1.028 | 0.973 |
| C4 | 1.031 | 0.970 |
| PLT | 1.056 | 0.947 |

VIF, variance inflation factor; PLT, blood platelet.

**Figure S1.** Histograms of plasma C4 levels among the HD patients. a. The histogram of plasma C4 levels among the whole cohort. b. The histogram of plasma C4 levels in the died and the alive groups.

**b**

**a**

**Figure S2.** Correlations between plasma levels of C4 and TG and HDL. **a.** Scatter plot for C4 and TG. Spearman’s rho=0.62, p<0.001. **b.** Scatter plot for C4 and HDL. Spearman’s rho=-0.38, p<0.001.

**a**

**b**

TG: triglyceride; HDL: high-density lipoprotein cholesterol.


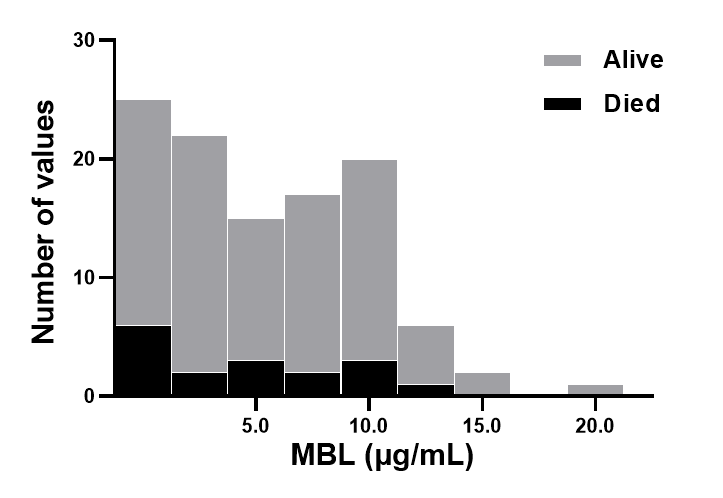


**Figure S3.** The histogram of plasma MBL levels in the died and the alive groups. There was a considerable amount of the death whose MBL level was lower than 2.5μg/ml [as 7 of 17 patients (41.2%)]

MBL, mannose-binding lectin.
